# Effects of Symptom Perception Interventions on Trigger Identification and Quality of Life in Children with Asthma

**DOI:** 10.1155/2015/825137

**Published:** 2015-10-28

**Authors:** Thomas Janssens, Andrew Harver

**Affiliations:** ^1^University of Leuven, Tiensestraat 102, Box 3726, 3000 Leuven, Belgium; ^2^The University of North Carolina at Charlotte, Charlotte, NC 28223, USA

## Abstract

*Background*. Management of individual triggers is suboptimal in practice. In this project, we investigated the impact of symptom perception interventions on asthma trigger identification and self-reported asthma quality of life. *Methods*. Children with asthma (*n* = 227) participated in three asthma education sessions and then were randomized first to one of three home monitoring conditions (symptom monitoring and peak flow training with feedback, peak flow training without feedback, or no peak flow training) and then subsequently to one of three resistive load discrimination training conditions (signal detection training with feedback, signal detection training without feedback, or no training). Triggers were reported at enrollment, following home monitoring, and following discrimination training; quality of life was measured after home monitoring and after resistive load testing. *Results*. Symptom perception interventions resulted in increases in reported triggers, which increased reliably as a function of home monitoring, and increased further in participants who completed discrimination training with feedback. Increases in the number of reported asthma triggers were associated with decreases in quality of life. *Discussion*. Patients may benefit from strategies that make trigger-symptom contingencies clear. Complementary strategies are needed to address changes in the perceived burden of asthma which comes from awareness of new asthma triggers.

## 1. Introduction

Indoor and outdoor allergens, intense emotion, irritants, physical exercise, and respiratory infections trigger asthma symptoms [1, 2], and both national and international asthma management guidelines emphasize the relevance of trigger knowledge and avoidance to achieve asthma control [3–6]. Although asthma triggers are often discussed with health care providers, trigger management remains suboptimal in clinical practice and individuals with asthma often report not knowing their triggers [5, 7–10].

The identification of idiosyncratic triggers by patients may be complex because relevant triggers are not always easy to determine and some triggers may be identified more readily than others [11]. Theoretically, accurate identification of asthma triggers results from three interacting processes: (a) identification of potential asthma triggers, (b) perception of asthma symptoms, and (c) recognition of a contingency between triggers and symptoms [11]. Trigger knowledge and avoidance may be hampered by inconsistencies in one or more of these processes and explain, in part, why patients with more severe or poorly controlled asthma report greater numbers of relevant asthma triggers [2, 12, 13].

Interventions to improve perception of symptoms may translate into improved perception of trigger-symptom contingencies. We evaluated the impact of two types of feedback-based interventions (peak flow monitoring and discrimination training) on both the frequency and the type of self-reported asthma triggers. Providing feedback for patient estimates of peak expiratory flow rate (PEFR) has been shown to increase perceptual accuracy of airflow obstruction and to improve adherence to inhaled corticosteroids [14, 15]; and feedback training for discrimination between the presence or absence of increases in the resistance to breathing has been shown to yield improved perception of airflow obstruction [16–18]. We examined not only the effects of these interventions on self-reported triggers but also the effects of changes in the number of perceived triggers on asthma quality of life [19, 20]. We hypothesized that changes in quality of life follow changes in the identification of relevant asthma triggers.

## 2. Materials and Methods

### 2.1. Research Setting and Participants

The data reported in this paper were collected within the context of Project On TRAC (Taking Responsibility for Asthma Control), a pediatric asthma research program. Program participants were between 8 and 15 years of age, were diagnosed with asthma at least two years prior to their entry into the study, were prescribed daily controller medications, and reported at least occasional asthma symptoms and/or nighttime cough.

At an initial enrollment session, children and their families were advised about all aspects of the research protocol, each child's legal guardian provided written informed consent, and the child provided written informed assent. Institutional review boards at both UNC Charlotte (#09-09-03) and Ohio University (#03F024) approved the research protocol. In addition to an enrollment session at which both children and caregivers completed baseline measures, the program consisted of three sessions of asthma education, two cycles of home monitoring of asthma symptoms for 30 days, four resistive load detection sessions, and six-month follow-up. This report provides detailed analysis of trigger identification and associated quality of life in children (*n* = 227) who completed data collection activities at enrollment, at the end of the first cycle of home monitoring, and at the end of resistive load testing.

Children were randomized to one of three home monitoring conditions at the end of the second asthma education session: PEFR training with feedback, PEFR training without feedback, and no PEFR. All conditions involved daily use of a common asthma symptom diary that included questions on symptoms, activity limitations, nighttime awakenings, and self-efficacy, as well as an open-ended question on perceived causes of symptoms. Additionally, children assigned to both PEFR training conditions recorded both estimated and actual PEFR values with the AM2+ Asthma Monitor (Jaeger, Hoechberg, Germany); the meters were programmed to display actual PEFR values to feedback group children but not to children in the no-feedback group. Effects of home monitoring on the correspondence between estimated and actual peak flow measures, and between peak flow measures and asthma symptoms, have been presented in preliminary form [16] and are the focus of a separate manuscript.

At the end of home monitoring, children completed one of three resistive load detection conditions: signal detection training with feedback, signal detection training without feedback, and no resistive load training. Children were assigned at random to one condition following completion of the first resistive load detection session, which established the threshold resistance to breathing in all participants [17]. Children assigned to the signal detection conditions determined whether or not an increased resistance to breathing was evident on selected breaths; children assigned to the feedback condition were given feedback regarding the accuracy of their responses whereas children assigned to the no feedback condition were kept uninformed about their performance. The effects of discrimination training on the perception of resistive loads were published previously [18].

### 2.2. Study Procedures

**Figure 1:**
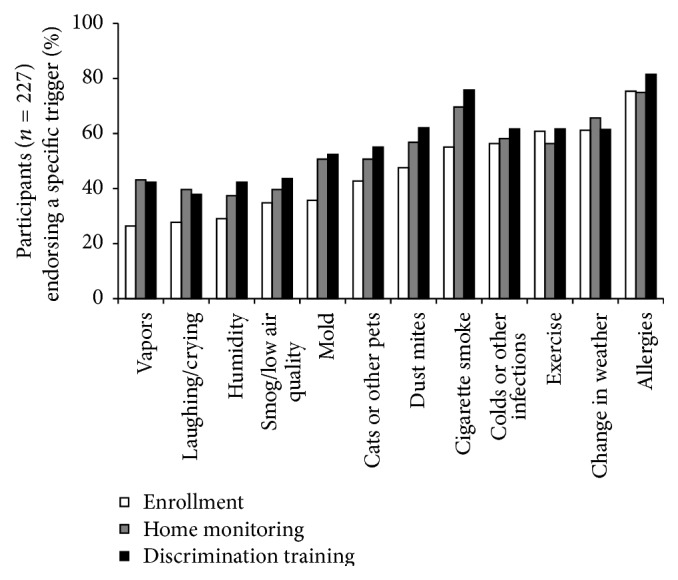
Trigger identification at enrollment, after home monitoring, and after discrimination training.

We collected participant demographic characteristics at enrollment and lung function was measured at the first asthma education session using the VMAX ENCORE 20C testing system (VIASYS Healthcare; Yorba Linda, California). Our primary outcome variables were asthma trigger identification and quality of life. At enrollment and following completion of both home monitoring and resistive load testing children were asked, “What usually triggers, or worsens, your asthma?” and they were instructed to select from a list of 12 possible triggers the ones that pertained to them and to add other items if necessary (Figure 1). Quality of life was measured with the Mini Asthma Quality of Life Questionnaire (Mini-AQLQ) [20] in children during the interview session (after home monitoring) and after resistive load testing.

At each interaction, children reported the degree to which they depended on symptoms for detecting worsening asthma (from not at all (“1”) to always (“10”)) and the extent to which they were able to predict an upcoming asthma attack (“yes” or “no”). We measured children's perceived asthma difficulty ranging from mild (“1”) to severe (“5”), from very well managed (“1”) to not managed at all (“5”), and from hardly noticeable (“1”) to very troublesome (“5”). We also conducted semistructured interviews with families at the end of home monitoring, which consisted of four open-ended questions about experiences and observations made by the child and family member during the home monitoring period.

### 2.3. Data Analysis

Data points were entered into our statistical program (SPSS version 22; IBM Inc.; Armonk, NY) and reviewed for accuracy. Data are presented as mean ± standard deviation unless otherwise noted. Differences in baseline data between groups were investigated using independent sample *t*-tests (*t*) or Chi-Square tests (*χ* ^2^); and we used analyses of variance (*F*) to test differences among groups. The total number of identified triggers and changes in specific triggers were analyzed using generalized hierarchical linear models using a 3 (peak flow monitoring condition: PEFR with feedback, PEFR without feedback, diary only) × 3 (resistive load testing condition: training with feedback, training without feedback, no training) × 3 (time: enrollment, after monitoring, after resistive load testing) design, and an unstructured covariance matrix. Items children added to our list of possible triggers (e.g., “grass,” “hay,” and “going to places”) were excluded from our analyses due to the low number of participants who responded (*n* = 9). Bonferroni corrections were used to control for multiple comparisons. SPSS employs Satterthwaite approximation for degrees of freedom, which we report rounded to the nearest integer.

Because the relationship between symptom perception and triggers is influenced by age, asthma duration, race, asthma control, and socioeconomic status (SES) [21, 22], we controlled for these variables in our analyses. Lung function (forced expiratory volume in one second percent predicted) and perceived asthma severity were used as indices of asthma control; levels of perceived asthma severity were related reliably to the number of reported asthma flare-ups the previous 12 months (*r*(216) = 0.19, *p* = 0.005) and to the number of school days missed the past year (*r*(223) = 0.34, *p* < 0.001). The Barratt Simplified Measure of Social Status was used to estimate SES, which is based on a weighted combination of education and occupation [23]. Caregivers reported the highest level of school completed by each parent and indicated their occupation from among clusters of related occupations that ranged from unemployed to physician, professor, or senior manager.

## 3. Results

### 3.1. Baseline Characteristics

A total of 227 participants completed both home monitoring and the resistive load training sessions. The sample included 155 boys and 72 girls, 98 were non-Hispanic Black and 112 were non-Hispanic White. Their age, on average, was 10 ± 1.6 years and children had been diagnosed with asthma for 6.6 ± 2.8 years. Average percent predicted lung function values for forced expiratory volume in one second (FEV_1_), forced vital capacity (FVC), and the FEV_1_/FVC ratio recorded at the first asthma education visit were 87.3 ± 19.1, 92.2 ± 17.4, and 82.5 ± 9.4, respectively.

At enrollment, participants selected, on average, 5.5 ± 2.7 triggers. The odds of identifying a trigger as relevant increased with more severe asthma for most triggers (9 of 12). Allergies were endorsed most often (75% of participants) and irritant vapors were endorsed least often (23% of participants; see Figure 1). Older participants were more likely to endorse exercise and humidity and less likely to endorse colds as relevant asthma triggers; those with higher SES were more likely to endorse allergens as asthma triggers, but less likely to endorse colds, mold, and weather changes as relevant (Table 1). Neither baseline characteristics nor the frequency of selected triggers at enrollment varied as a function of either home monitoring or discrimination training group assignment (data not shown).

### 3.2. Change in Trigger Identification

**Figure 2:**
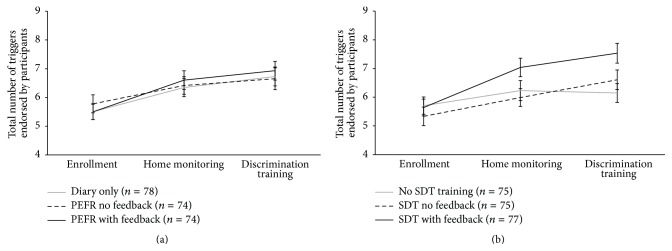
Total number of reported asthma triggers (mean ± SEM) for each home monitoring condition (a) and for each resistive load signal detection training (SDT) condition (b) at enrollment, after home monitoring, and after discrimination training.

The total number of triggers endorsed by children significantly increased over time (*F*(2,223) = 20.91, *p* < 0.001) both from enrollment to home monitoring (*t*(223) = 4.72, *p* < 0.001) and from home monitoring to discrimination training (*t*(222) = 2.90, *p* = 0.012) (Figure 2). The results of the covariate analyses demonstrated that greater numbers of new triggers were associated with lower SES (*F*(1,223) = 4.91, *p* = 0.028), longer duration of asthma (*F*(1,223) = 6.68, *p* = 0.01), and greater perceived asthma severity (*F*(1,223) = 20.77, *p* < 0.001), but not with participant age, sex, race, or lung function.

Increases in the number of triggers were not differentially affected by home monitoring condition (time × monitoring, *F*(4,223) = 0.43, *p* = 0.788). Interviews conducted at the end of the home monitoring period, however, consistently affirmed that families reported greater understanding of individual triggers. We provide the following examples of asthma trigger-related comments obtained during interviews held with families at the end of home monitoring in response to the question, “Did you learn anything from keeping the diary?”

Parent comments were as follows:

- We didn't know what triggered the girls' asthma so it helped us know that. That what we thought was congestion from a cold…was really from a trigger. We are on the right meds now.
- Learned the triggers, looking back through the day what triggers his physical symptoms.
- Helped me pin point his triggers which felt worse. Helped me to be more aware of his triggers.
- She's a bit more sensitive (to triggers) than I thought she was.
- I think she is more aware of her triggers since the program.
- Yes, we learned what his triggers were.

Child comments were as follows:

- How to control it more, I noticed triggers.
- Playing outside in the cold, being dehydrated (triggers that he didn't know before).
- Triggers: cold air, cats.
- To stay away from your triggers. When outside breathe through nose instead of mouth.
- It made me think about the triggers.

Resistive load training, on the other hand, resulted in differential increases in reported triggers (*F*(4,223) = 3.33, *p* = 0.011); participants who completed signal detection training with feedback subsequently reported more triggers compared to those who did not receive feedback training (*t*(226) = 2.91, *p* = 0.012).

**Figure 3:**
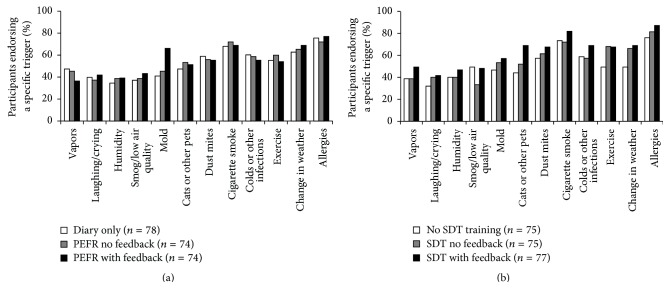
Triggers endorsed following home monitoring (a) and following resistive load signal detection training (SDT) (b).

Symptom interventions involving feedback differentially affected the perceived relevance of specific triggers (Figure 1). Participants assigned to peak flow monitoring with feedback were more likely to endorse mold as a trigger compared to other home monitoring conditions (Figure 3(a)); participants assigned to the signal detection training with feedback condition evidenced reliable increases in the identification of pets as relevant (Figure 3(b)).

### 3.3. Trigger Identification and Quality of Life

Regardless of feedback training experience, participants demonstrated overall improvement in quality of life, as well as improved self-reported asthma difficulty and ability to predict an asthma episode (Table 2). However, both the number of asthma triggers at enrollment and changes in asthma trigger identification had an impact on quality of life. At enrollment, participants who reported greater numbers of triggers not only evidenced a greater dependence on symptoms to manage asthma (*t*(255) = 2.26, *p* = 0.025) but also reported more severe, less well-managed, and more troublesome asthma as well as reduced asthma-related quality of life (Table 2).

Increases in the number of reported triggers across time were also associated with reduced asthma-related quality of life for overall mini-AQLQ scores life (*t*(415) = −6.55, *p* < 0.001) as well as for scores for each subscale (Table 2). We evaluated mini-AQLQ scores obtained after home monitoring between children who reported the same or fewer numbers of triggers compared to enrollment (*n* = 103) and those who reported one or more new triggers (*n* = 124). Reliably poorer quality of life scores were observed in children who reported more triggers for overall mini-AQLQ scores (*t*(225) = 3.05, *p* = 0.003) as well as for the subscales symptoms (*t*(225) = 3.16, *p* = 0.002), environment (*t*(225) = 3.47, *p* < 0.001), and emotions (*t*(225) = 1.99, *p* < 0.05). Similar findings were obtained for analyses conducted between children who reported the same or fewer numbers of triggers after discrimination training compared to those after home monitoring (*n* = 130) and those who reported one or more new triggers (*n* = 96). Reliably poorer quality of life was observed in children who reported more triggers for overall mini-AQLQ scores (*t*(224) = 2.14, *p* < 0.05) as well as for the symptom subscale (*t*(224) = 2.66, *p* = 0.008).

## 4. Discussion

The aim of this study was to evaluate the differential impact of two symptom perception interventions on asthma trigger identification and associated asthma quality of life in children with persistent asthma. Both the number and the types of triggers reported by participants at enrollment were similar to those observed by other groups [9, 12, 24, 25] and affirmed the variability in trigger prevalence reported in previous investigations [2, 10, 12, 24]. We observed that interventions involving accurate detection of airflow obstruction were effective at increasing trigger identification. Not all triggers were endorsed equally in this regard; those that were commonly reported at enrollment (e.g., allergies, weather, colds, and smog) were less influenced by our interventions. Feedback training experiences differentially affected the perceived relevance of specific triggers including mold and pets, triggers less likely discussed in encounters with health care professionals [8].

Increases in the number of triggers endorsed as relevant were not differentially affected by home monitoring condition. We attribute this uniform effect to daily use of an asthma symptom diary by all participants. Self-management is organized around the monitoring of disease-related variables, and diaries play a key role in establishing relationships between the environment and behavior in individual patients [26]. Despite widespread use of asthma diaries, daily recordings of triggers are not commonly evident in previous work [12]. Participants who completed resistive load discrimination training with feedback subsequently reported more triggers compared to those who did not receive feedback training. Taken together, interventions aimed at facilitating perception of airflow obstruction may serve to reinforce associations among disease-related variables, including symptom-trigger contingencies, and may be beneficial in discerning problematic sources of symptoms [11, 26].

Participants who reported greater numbers of triggers also reported lower levels of asthma-related quality of life. Although these findings may appear counterintuitive, they might be expected based on earlier work. First, new triggers may result in increases in the perceived burden of asthma in accordance with other studies that showed associations between the number of perceived asthma triggers and poorer asthma outcomes [12, 27]. Our finding that increases in trigger identification were associated with an increased reliance on symptoms to guide asthma management corroborates this line of thinking. Second, patients may have concluded that changes in asthma self-management behaviors to avoid or reduce trigger exposures were ineffective [10, 15, 28]. Trigger knowledge does not automatically lead to adequate trigger avoidance or removal, as studies have shown that exposure to known allergens and other environmental triggers can remain high [5, 25]. Third, avoiding or managing new triggers may result in behavioral adjustments (e.g., staying indoors) that negatively impact health-related quality of life, effects that have not been well documented [1, 5, 7, 11, 12, 24]. Trigger education research is needed to address effective self-management strategies that reduce the perceived burden of asthma when new asthma triggers are discovered.

Our observations are in contrast to the positive effects of extensive environmental interventions on health outcomes in children with asthma, implemented in mostly urban settings and focused primarily on minority populations [29–32]. Our sample, for example, was neither inner city nor disadvantaged; and similar proportions of black and white children were prescribed either inhaled corticosteroids with or without a long-acting beta-agonist (73% and 79%, resp.) and/or leukotriene modifiers (57% and 60%, resp.). On the other hand, covariate analyses showed that lower SES, longer asthma duration, and increased perceived asthma severity at enrollment were associated with greater increases in reported asthma triggers over time. These findings are consistent with a large body of work highlighting differences in both trigger knowledge and exposure between low-SES and high-SES individuals [33, 34]. Subsequent research may clarify the importance of trigger awareness interventions tailored to the needs of particular groups of patients.

Limitations are evident in our approach. We were unable to confirm directly the effects of existing or newly identified asthma triggers on asthma control or to differentiate between increases in the identification of previously unknown triggers from improvements in trigger identification accuracy. On the other hand, self-monitoring involving use of a daily diary appears as an especially feasible approach for confirming symptom-inducing properties of allergic as well as physical and environmental asthma triggers. Second, we assessed changes in quality of life immediately following the conclusion of symptom monitoring interventions; our design precluded assessment of possible long-term benefits of trigger awareness. Third, the types of triggers we measured were similar to those in inventories employed in previous investigations, although our inventory was relatively lacking in psychological triggers [35]. Recent efforts have been made to collect trigger data in standardized ways [11, 12], which may benefit future research on trigger identification [1] as well as routine documentation of asthma triggers in clinical practice [36]. Finally, all participants received education and completed symptom diaries, which precludes us from evaluating the independent contributions of these two activities on asthma trigger identification.

Although many asthma triggers are modifiable risk factors, trigger avoidance advice is not universal in practice and efforts to control trigger exposures are not uniformly effective [6, 8–10, 12, 24, 36]. We have demonstrated that participants randomized to symptom perception interventions that included feedback training for accurate detection of airflow obstruction report increases in the relevance of specific triggers. Such interventions may reinforce associations between disease-related variables, including symptom-trigger contingencies, and contribute to increased trigger awareness. However, the association of increased trigger reports with poorer quality of life suggests that additional actions may be required of patients to confront the burden of newly identified asthma triggers.

## 5. Conclusions

Effective asthma management includes not only assessment and monitoring of asthma control, education that enables patient-provider asthma partnerships, and adequate pharmacotherapy but also tailored trigger knowledge and avoidance [4]. Interventions involving accurate detection of airflow obstruction are effective at increasing trigger identification and may serve to reinforce associations between disease-related variables including symptom-trigger contingencies. Increases in the perceived burden of asthma which comes from awareness of new asthma triggers, however, may complicate management goals set by health care providers.

## Figures and Tables

**Table 1:** Associations between covariates and individual triggers.

Covariate	Trigger	OR	Lower 95% CI	Upper 95% CI	*t*	*p*
Age	Exercise	1.21	1.02	1.42	2.21	0.028
	Humidity	1.23	1.05	1.44	2.52	0.012
	Colds	0.83	0.72	0.96	−2.57	0.011

Asthma duration	Weather	1.14	1.03	1.25	2.58	0.011

Race (White versus Black)	Exercise	2.05	1.01	4.04	2.44	0.047

Sex (female versus male)	Cigarette smoke	2.06	1.15	3.70	2.45	0.015

SES	Allergies	1.01	1.00	1.02	2.13	0.034
	Colds	0.99	0.98	1.00	−2.03	0.044
	Mold	0.99	0.98	1.00	−2.22	0.028
	Weather	0.99	0.98	1.00	−2.66	0.008

Perceived severity	Laughing/crying	1.25	1.03	1.54	2.21	0.028
	Humidity	1.33	1.10	1.62	2.94	0.004
	Dust Mites	1.29	1.07	1.57	2.63	0.009
	Cigarette smoke	1.44	1.17	1.78	3.42	0.001
	Colds	1.02	1.01	1.42	2.07	0.04
	Mold	1.28	1.05	1.57	2.47	0.014
	Smog	1.50	1.25	1.81	4.31	<0.001
	Vapors	1.42	1.16	1.74	3.37	0.001
	Weather	1.35	1.10	1.65	2.95	0.004

OR = odds ratio (odds of reporting a trigger/odds of not reporting a trigger), adjusted for covariates; CI = confidence interval; *t* = *t*-statistic; *p* = probability; SES = socioeconomic status.

**Table 2:** Self-reported asthma outcomes and associations with asthma trigger identification.

Variable	Enrollment		Home monitoring		Discrimination training				* * Association with trigger identification at enrollment			Change		
	Mean	SD	Mean	SD	Mean	SD	*F*	*p*	Estimate	SE	*p*	Estimate	SE	*p*
Self-reported asthma difficulty														
Severe	2.59	1.21	2.33	1.20	2.28	1.24	9.69	<0.001	0.11	0.02	<0.001	0.07	0.02	<0.001
Managed	2.71	1.07	2.37	1.11	2.20	1.04	22.99	<0.001	0.04	0.02	0.037	0.05	0.02	0.006
Troublesome	2.85	1.16	2.67	1.06	2.50	1.03	12.20	<0.001	0.10	0.02	<0.001	0.05	0.02	0.002
Depend on symptoms	6.41	2.70	5.26	2.88	5.69	3.02	13.87	<0.001	0.09	0.05	0.077	0.12	0.05	0.022
Able to predict an attack (*n*, %)	109	48%	111	50%	131	58%	4.62	0.01	0.082	0.05	0.079	−0.01	0.04	0.795
Asthma-related quality of life														
Symptoms			4.90	1.51	5.03	1.44	4.25	0.04	−0.14	0.03	<0.001	−0.16	0.03	<0.001
Activities			5.81	1.37	6.04	1.27	7.57	0.006	−0.15	0.03	<0.001	−0.14	0.02	<0.001
Emotions			5.29	1.53	5.57	1.49	11.58	0.001	−0.12	0.03	0.001	−0.08	0.03	0.004
Environment			4.78	1.74	5.02	1.65	8.72	0.003	−0.22	0.04	<0.001	−0.19	0.03	<0.001
Overall			5.19	1.30	5.40	1.21	12.93	<0.001	−0.15	0.03	<0.001	−0.14	0.02	<0.001

Asthma difficulty was rated from mild (‘‘1”) to severe (‘‘5”), from very well managed (‘‘1”) to not managed at all (‘‘5”), and from hardly noticeable (‘‘1”) to very troublesome (‘‘5”). SD = standard deviation; *F* = *F*-test; *p* = probability; SE = standard error.
